# Case report: Chest radiotherapy-induced vertebral fractures in lung cancer patients: a case series and literature review

**DOI:** 10.3389/fonc.2025.1438120

**Published:** 2025-02-03

**Authors:** Oscar Arrieta, Francisco Lozano-Ruiz, Alberto Guijosa, Pamela Soberanis-Pina, Monika Blake-Cerda, Ana Pamela Gómez-García, Federico Maldonado-Magos, Emilio Conde-Flores, Andrés F. Cardona, Sandra Ileana Pérez Álvarez, Luis Antonio Cabrera-Miranda

**Affiliations:** ^1^ Thoracic Oncology Unit, Departamento de Oncología Torácica, Instituto Nacional de Cancerología (INCan), Mexico City, Mexico; ^2^ Radiotherapy Unit, Instituto Nacional de Cancerología (INCan), Mexico City, Mexico; ^3^ Radio-Oncology Unit, Cancer Center ABC, Medical Center, Mexico City, Mexico; ^4^ Service of Anatomical Pathology, Medica Sur Clinic & Foundation, Mexico City, Mexico; ^5^ Direction of Research and Education, Luis Carlos Sarmiento Angulo Cancer Treatment and Research Center - CTIC, Bogotá, Colombia

**Keywords:** fracture, radiotherapy, toxicity, lung cancer, long-term survivors

## Abstract

**Background:**

As survival rates for lung cancer (LC) patients continue to rise, the adverse impacts of therapies become more relevant. Radiotherapy is known to negatively affect bone health. However, radiotherapy-induced vertebral fractures in lung cancer patients remain an exceedingly rare and underrecognized condition that could be mistaken for bone metastasis.

**Case presentation:**

We identified three LC patients (all long-term survivors), aged 67 to 81, who developed thoracic vertebral fractures post-chest radiotherapy, within irradiated fields; two had advanced non-small cell lung cancer (NSCLC) and one had extensive small cell lung cancer (SCLC). Baseline imaging confirmed that the fractures occurred after therapy. The median time from radiotherapy to fracture onset was 19 months (range: 1-30 months), with a median follow-up time from the initial fracture of 39 months (range: 37-61 months). All observed fractures were compressive in nature. These patients shared common characteristics, including advanced age, a history of heavy smoking, and high radiation doses. Additionally, hypermetabolic activity at the fracture sites necessitated MRI to differentiate these fractures from bone metastases. Management involved interventional strategies such as vertebroplasty, kyphoplasty, and rhizotomy, along with general and pharmacological measures to prevent subsequent fractures.

**Conclusions:**

Despite their low incidence, radiotherapy-induced vertebral fractures in LC patients are clinically significant and may resemble bone metastases on PET-CT imaging. MRI, alongside risk factors similar to those of osteoporosis, can facilitate prompt identification and differentiation. As survival rates in LC patients improve, the relevance of this adverse effect increases, underscoring the need for implementing bone protective strategies to further enhance patient outcomes and quality of life.

## Introduction

Radiotherapy, a cornerstone in cancer treatment, is pivotal in improving survival and quality of life for lung cancer (LC) patients. Nonetheless, the recent emergence of novel therapeutics, which have significantly extended life expectancy among LC patients, has highlighted the long-term deleterious effects of radiotherapy. Previously considered infrequent, these adverse impacts are now increasingly common and significant (1).

An important, yet scarcely researched phenomenon, is the detrimental effect of radiotherapy on bone health. It has been established that radiotherapy leads to osteopenia (2); nonetheless, the subsequent and less commonly recognized issue is radiotherapy-induced fractures, which may manifest and reverberate in a more long-term context. Specifically, there is a documented increase in the incidence of pathological fractures, ranging from 1.2% to 25%, in patients undergoing radiotherapy. These fractures are most commonly observed in the ribs, pelvis, and femur (3), though the incidence may vary significantly based on clinical characteristics and radiation factors such as dosage and fractionation (4).

Owing to its low incidence, little is known about the clinical characteristics of patients with radiotherapy-induced vertebral fractures following chest radiotherapy. Such fractures can be easily mistaken for metastatic bone disease progression or may be overlooked and inadequately treated. Additionally, there is no established dose at which these fractures occur, and consequently, no dose constraints to the radiation that bone should receive have been established, as there have with other structures (e.g., spine) (5). Accordingly, proposals for general and pharmacological strategies to safeguard bone health in these patients are lacking.

This work reports three unique cases of LC patients who developed radiotherapy-induced vertebral fractures following chest, accompanied by a brief review of the literature on this topic. To our knowledge, this is the inaugural case series addressing this rare but significant phenomenon.

## Case description

### Case 1

**Figure 1:**
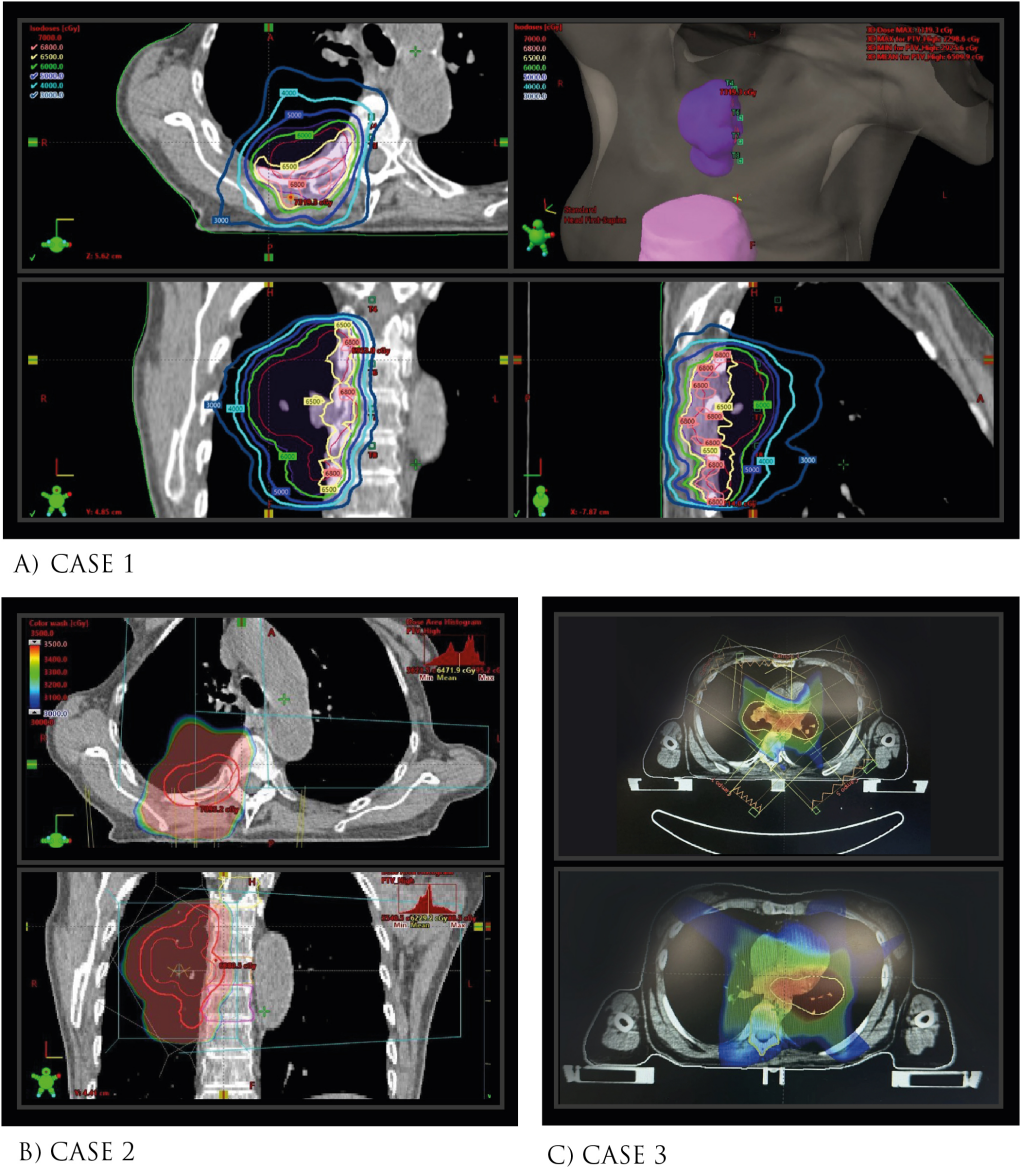
Schematic of Radiation Dose Administered and Planning in Pre-Radiotherapy Chest CT. **(A)** Case 1 **(B)** Case 2, **(C)** Case 3.

An 81-year-old woman with a 20-pack-year smoking habit, previously treated for breast cancer in 2004 (quadrantectomy and adjuvant chemoradiotherapy) and gastric cancer in 1988 (subtotal gastrectomy) with trauma history only relevant for an ankle fracture treated with open reduction and internal fixation. She was diagnosed in 2019 with Stage IVA(T3N2M1a) poorly-differentiated solid adenocarcinoma of the lung, exhibiting high PD-L1 expression (90%). Initial PET-CT scan showed a 55x43 mm mass in the right lower lobe with significant pleural thickening of 15mm and an SUVmax of 7.6, alongside metabolic activity in the mediastinal lymph nodes. She initiated treatment with carboplatin, pemetrexed, and pembrolizumab, later transitioning to pembrolizumab monotherapy due to interstitial nephritis. After three cycles, a PET-CT in January 2020 revealed a partial response. She underwent radiotherapy to the primary lesion (60 Gy in 30 fractions) from March to May 2020 (**Figure 1A**).

**Figure 2:**
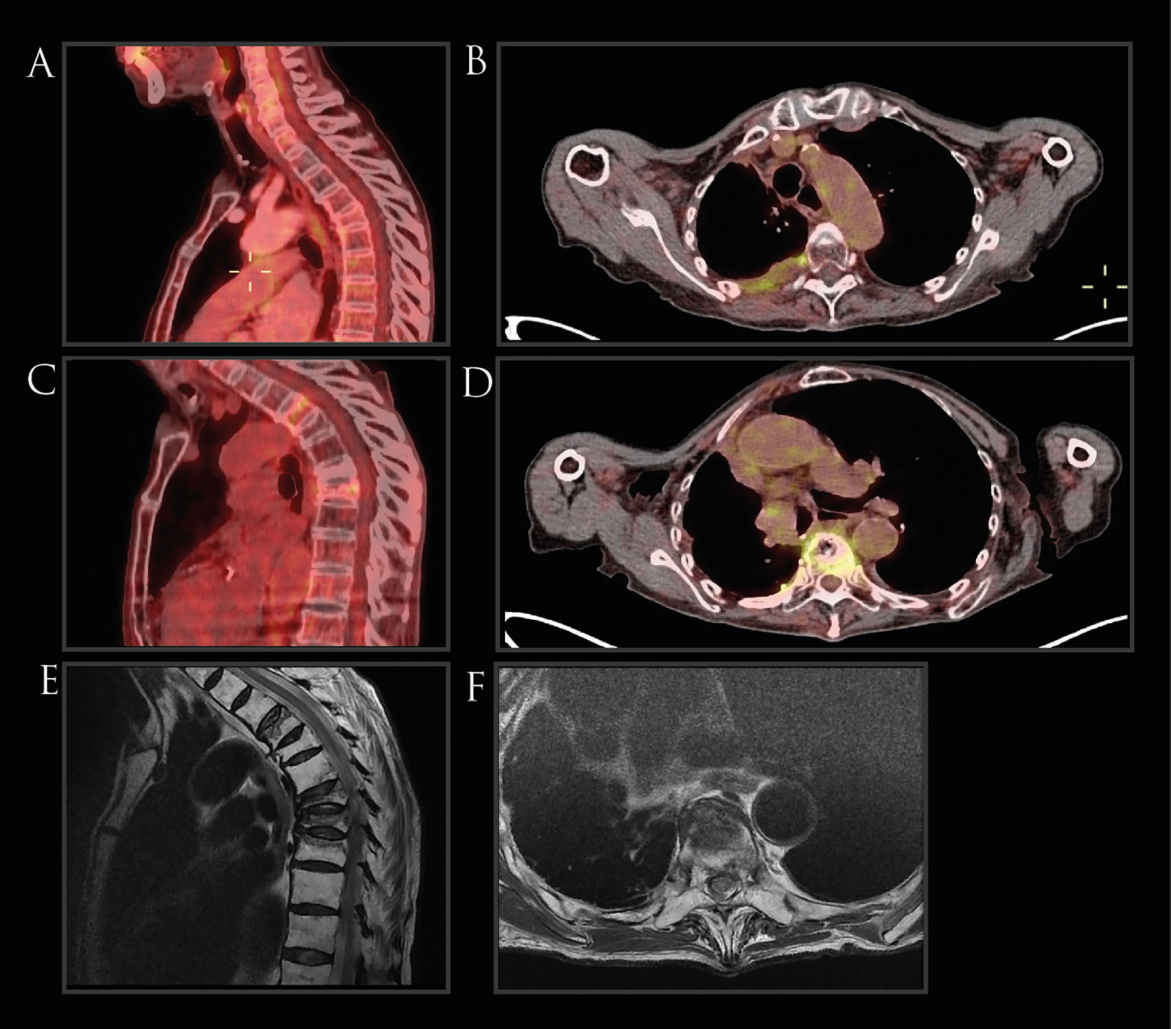
Imaging of Case 1. **(A, B)** PET-CT scans conducted prior to radiotherapy revealed no evidence of vertebral fractures. **(C, D)** Follow-up PET-CT scans post-radiotherapy, depicting compression fractures in the T4, T7, and T8 vertebrae, accompanied by diffuse metabolic activity, absent in prior imaging. **(E, F)** MRI showing the presence of vertebral fractures.

Later, a January 2021 PET-CT disclosed new compression fractures at the T4, T7, and T8 vertebral levels, associated with diffuse metabolism but without tumor evidence. At the time, the patient did report dull back pain but no history of trauma. MRI was ordered, which showed an anterior third collapse in the vertebral bodies T4 and T6-T9 (all within the irradiated field; **Figure 1A**), consistent findings with insufficiency fractures (**Figure 2**). Treatment involved dorsal epiduroscopy and vertebroplasty with radiofrequency rhizotomy for pain management. At 86, she uses analgesics and a corset for back pain, which has been well controlled, showing no tumor activity to date (39 months after the vertebral fractures).

### Case 2

A 77-year-old male with a 22.5-pack-year smoking history, hypertension, COPD, an 18.6 BMI, and no trauma history was diagnosed with stage IIIA(T2aN2M0) moderately differentiated adenocarcinoma of the lung. Initial July 2017 PET-CT revealed a 37-mm spiculated lesion in the parahilar region of the right upper lobe, and mediastinal lymphadenopathies at levels 4R and 7R. After three cycles of neoadjuvant carboplatin-pemetrexed, he underwent a right lung lobectomy and mediastinal lymph node dissection, revealing significant treatment response with minimal residual lesion (0.2 cm) and lymph node hyperplasia. Post-surgery, he completed three more chemotherapy cycles.

**Figure 3:**
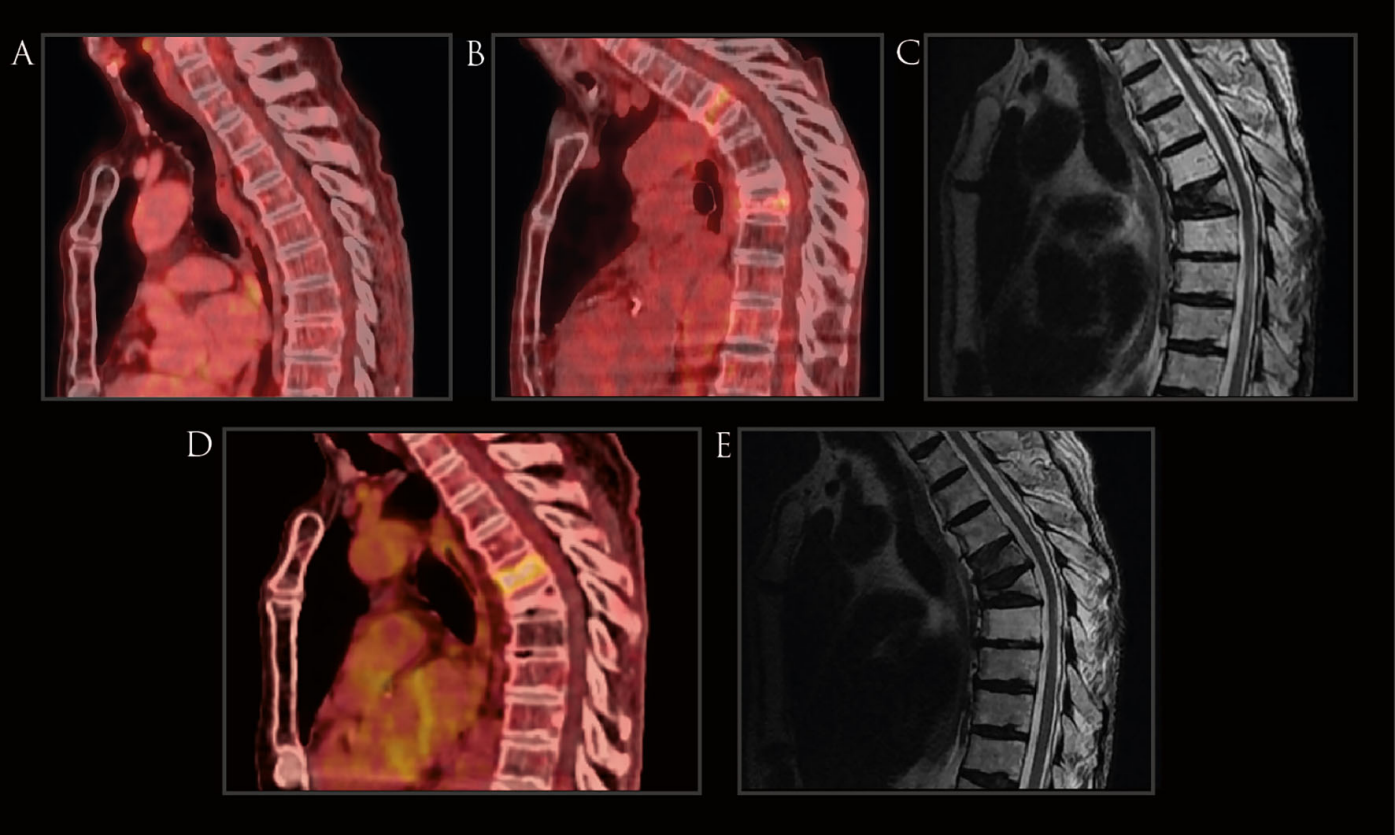
Imaging of Case 2. **(A)** PET-.CT scans conducted prior to radiotherapy revealed no evidence of vertebral fractures. **(B)** Follow-up PET-CT scans post-radiotherapy, depicting a compression fracture in the T7 vertebra, accompanied by diffuse metabolic activity, absent in prior imaging. **(C)** MRI showing the presence of the T7 vertebral fracture. **(D)** Follow-up PET-CT scan depicting new compression fracture in the T6 vertebra, accompanied by diffuse metabolic activity **(E)** MRI showing the presence of T6 and T7 compression fractures with anterior wedging.

A PET-CT in April 2018 identified a new hypermetabolic lymph node at level 4R, leading to mediastinal radiotherapy (66 Gy in 33 fractions) from June to August 2018 (**Figure 1**). Subsequent PET-CT in September 2018 revealed a decrease in mediastinal adenopathy metabolism, but a hypermetabolic vertebral compression fracture at T7 (SUVmax 3.12), confirmed by MRI to be inflammatory rather than neoplastic (**Figure 3**). The patient did report that for the past month, he had had moderate back pain but no history of falls. Management included a corset and T7 vertebroplasty in February 2019 which controlled the pain. In April 2019, he started biannual intravenous alendronate treatment.

Oncologically, he remained under surveillance until a February 2021 PET-CT detected focal hypermetabolism in the T6 vertebral body with anterior wedging (SUVmax 4.1), a new finding compared to previous scans. The tumor multidisciplinary board reviewed the case, recommending an MRI for further characterization of the hypermetabolic area. Depending on the results, the lesion would either be treated with radiation for suspected metastatic disease or referred to orthopedics if metastasis was ruled out. The MRI in May 2021 identified a subsequent subacute compression fracture in the T6 vertebral body without evidence of metastatic disease. Additionally, low vitamin D levels were detected, leading to the initiation of calcium/vitamin D supplementation to which the patient was well adherent. At age 83 the patient remains asymptomatic with no signs of active tumor.

### Case 3

A 67-year-old female with a 40-pack-year smoking history presented with left facial swelling and cough. October 2014 PET-CT demonstrated extensive lymphadenopathy involving cervical, mediastinal, hilar, and interlobar regions. Biopsy confirmed small-cell lung carcinoma. Brain MRI revealed no metastases. Treatment began with two cycles of cisplatin/etoposide chemotherapy, followed by concurrent chemoradiotherapy (45Gy in 30 [150cGy] fractions BID; **Figure 1C**) with cisplatin for four weeks and prophylactic cranial irradiation. This was succeeded by four cycles of consolidation adjuvant chemotherapy with cisplatin/etoposide from January 7 to April 27, 2015. Subsequent PET-CT showed a complete response, and the patient remained under surveillance.

In July 2017, a non-contrast chest CT revealed a T8 vertebral fracture, with MRI confirming it as an anterior wedging compression fracture (**Supplementary Figure 1**). The patient was asymptomatic, with no pain or other acute complaints. A PET-CT scan found no metabolic indications of tumor activity. The patient underwent T8 kyphoplasty in October 2017 and was discharged. At age 73, she continued under surveillance with no disease evidence, and without major fracture symptoms.

## Relevant patient data

Relevant patient data from the three LC cases with radiotherapy-induced vertebral fractures are shown in **Table 1**.

**Table 1:** Clinical features of patients with radiotherapy-induced vertebral fractures.

	Case No.			
	Case 1	Case 2		Case 3
Age, years	81	77		67
Sex	Female	Male		Female
Performance status, ECOG	1	1		1
Risk factors	Former heavy smoker Adjuvant RT for breast cancer in 2004 Impaired Renal Function	Former heavy smoker Low BMI Vitamin D Deficiency COPD		Former heavy smoker
Histologic type	Poorly-differentiated solid adenocarcinoma	Moderately-differentiated solid adenocarcinoma		Small-cell lung carcinoma
Stage	IVA	IIIA		Extensive
Tumor location	Right lower lobe	Right parahilar		Right
Initial treatment	CBP/pemetrexed + pembrolizumab (9 cycles)	Surgery + adjuvant CBP/pemetrexed		Cisplatin/Etoposide
RT Regimen	60Gy in 30 fractions	66Gy in 33 fractions		45Gy in 30 fractions
Dose per fraction	2 Gy	2 Gy		1.5 Gy
RT to adjacent vertebra w/o malignancy	03.2020-05.2020	06.20-08.2018		11.2014-01.2015
	Sequential	Sequential		Concurrent and sequential
Vertebrae w/fracture	T4, T6, T7, T8 and T9	T7	T6	T8
Fracture Date	01.2021	09.2018	02.2021	07.2017
Time from RT to fracture	8 months	1 month	30 months	30 months
Minimum Vertebral Radiation Dose (cGy)	5220	5520		1680
Mean Vertebral Radiation Dose (cGy)	6556	7006		3210
Maximum Vertebral Radiation Dose (cGy)	7169	7481		4480
Type of fracture	Compression fracture	Compression fracture		Compression fracture
Fracture treatment	Vertebroplasty + Rhizotomy	Vertebroplasty	Conservative	Kyphoplasty

ECOG, Eastern Cooperative Oncology Group; RT, Radiotherapy; BMI, Body Mass Index; COPD, Chronic Obstructive Pulmonary Disease.

## Discussion

The presented case series examines three lung cancer patients who developed vertebral fractures post-chest radiotherapy, all confined within irradiated areas and with bone metastasis radiologically excluded. Importantly, all presented cases are confirmed to remain disease-free to date, highlighting the necessity of early identification and differentiation of this entity from disease progression.

Although not fully understood, the mechanisms of radiation-induced bone damage encompass direct injury from ionizing radiation via free radicals and inflammation, osteoclast-driven resorption, and vascular damage leading to hypoxia, which impairs bone regeneration, increasing the risk of osteopenia and consequent fractures (6). Cancer patients are particularly susceptible to bone loss, as they are often older and undergo therapies detrimental to bone health. Chemotherapeutic agents, like cisplatin, decrease calcium levels, while aromatase inhibitors used in breast cancer treatment lead to bone loss. Furthermore, the proinflammatory nature of cancer itself alters the bone microenvironment, promoting increased bone turnover (7).

In LC, knowledge of radiotherapy’s impact on bone health primarily comes from studies on stereotactic body radiotherapy (SBRT), associated with higher *rib fracture* risk than conventional radiotherapy, likely from hypofractionated doses indicating a dose-volume link to bone loss (8). Conversely, *vertebral fractures* from conventional chest radiotherapy are significantly less common. Consequently, this type of fracture may be clinically unrecognized and mistaken for bone metastasis, leading to erroneous treatment decisions, such as unnecessary systemic therapy for presumed recurrent/progressive disease, or additional radiotherapy which would exacerbate the condition.

In this scenario, PET-CT, often revealing increased activity in post-fracture bone changes, may not suffice for differential diagnosis, necessitating additional diagnostic approaches, including detailed clinical assessments, advanced imaging, or even biopsy (9). Our series suggests that MRI is particularly useful in identifying insufficiency fractures caused by radiation, which often present as compression fractures. This aligns with the reported sensitivity and specificity of MRI (95.3% and 92.8%, respectively) in distinguishing insufficiency fractures from bone metastases, where it has consistently been shown to be useful (10).

Moreover, our cases underscore the need for precise assessment of irradiated areas and doses; vertebrae in two patients received mean ~65-70 Gy and developed fractures, whereas another patient fractured at roughly half the dose (32 Gy), likely due to the higher biologically effective dose (BED) from twice-daily SCLC radiotherapy (BED for α/β=3 is 47 Gy). In line with recent evidence linking high-dose thoracic vertebrae irradiation to acute hematologic toxicities in LC patients, our findings support adopting bone-sparing radiation strategies (11).

Research across various cancers has shown that factors like female sex, advanced age, low BMI, and chemotherapy exposure elevate fracture risk (12). Although data for LC patients are limited, this case series suggests that risk factors for radiotherapy-induced vertebral fractures might resemble those associated with osteoporosis. LC patients may be particularly vulnerable, given reports that up to a third of them suffer from osteoporosis and they commonly harbor additional risk factors for fracture (13, 14).

Consistent with previous findings, advanced age — associated with increased rates of osteopenia and osteoporosis, as well as significant comorbidities — emerges as a possible risk factor (12, 13). Furthermore, the commonality of smoking in LC patients, which is linked to a 5-10% reduction in bone density, may increase risk (15). This was observed in our series, where all participants were heavy smokers. Additional clinical factors, like a history of malignancy or prior radiation, should also heighten concern for increased risk.

A single case report has been published describing one LC patient who developed a vertebral fracture after receiving conventional chest radiotherapy (16). Additionally, two abstracts on radiotherapy-induced vertebral fractures following chest radiotherapy have been published, one involving conventional radiotherapy (17) and the other SBRT (18). Despite the limited information in the latter two reports, they collectively suggest high radiation doses, female sex, and older age as potential risk factors. Moreover, these cases highlight the importance of osteopenia in the risk of fracture, which was not measured in our cases. The characteristics of these patients are included in **Supplementary Table 1**.

Given the risk of bone loss and fractures in LC and radiotherapy, and the growing impact of skeletal events on quality of life as survival increases (19), integrating preventive measures into comprehensive cancer care plans is crucial. A skeletal health program, akin to those for osteoporosis management, is recommended for all patients. This includes dietary counseling to ensure optimal calcium (1200 mg/day) and vitamin D (800 IU/day) intake for all patients, and supplementation for those unable to meet these requirements. Additionally, evidence supports the involvement of patients in physical therapy for muscle strengthening, as well as smoking cessation efforts and management of comorbidities that may elevate fracture risk (20).

Pharmacological interventions for fracture prevention should be customized to the individual’s fracture risk profile, incorporating both bone mineral density and clinical risk factors. Bisphosphonates, which counteract early radiation-induced bone collagen degradation, have shown effectiveness in managing skeletal metastases in patients receiving radiotherapy. Similarly, denosumab has been demonstrated to delay skeletal-related events in this demographic. Therefore, combining any of these therapies with radiotherapy may be advisable for patients at increased risk (21–23).

For patients who experience bone fractures, the focus should shift to effective pain management and preventing future fractures. Initial measures should include strategies such as promoting early mobility, implementing bed rest, and applying bracing, in conjunction with a medication regimen that includes over-the-counter analgesics or opioids. If these approaches are insufficient, vertebroplasty or kyphoplasty may be considered for significant pain relief and improved quality of life (24).

## Conclusion

This case series recognizes radiotherapy-induced vertebral fractures as an important adverse impact in LC patients an underscores the crucial need for their differentiation from bone metastases, highlighting the utility of MRI for diagnosis. It advocates for comprehensive bone-protective measures for all patients and pharmacological interventions for at-risk individuals, aiming to enhance patient care and quality of life post-radiotherapy in this often unrecognized condition. The significance of these considerations escalates with the increasing lung cancer survival rates.

## Data Availability

The original contributions presented in the study are included in the article/**Supplementary Material**. Further inquiries can be directed to the corresponding author.

## References

[1] Or M Liu B Lam J Vinod S Xuan W Yeghiaian-Alvandi R. A systematic review and meta-analysis of treatment-related toxicities of curative and palliative radiation therapy in non-small cell lung cancer. Sci Rep. (2021) 11:5939. doi: 10.1038/s41598-021-85131-7 33723301 PMC7971013

[2] Wissing MD. Chemotherapy- and irradiation-induced bone loss in adults with solid tumors. Curr Osteoporos Rep. (2015) 13:140–5. doi: 10.1007/s11914-015-0266-z PMC4417126 25712619

[3] Soares CBG Araujo ID Padua BJ Vilela JCS Souza RHR Teixeira LEM. Pathological fracture after radiotherapy: systematic review of literature. Rev Assoc Med Bras (1992). (2019) 65:902–8. doi: 10.1590/1806-9282.65.6.902 31340323

[4] Fujii K Sakanaka K Uozumi R Ishida Y Inoo H Tsunoda S. Association of chemoradiotherapy with thoracic vertebral fractures in patients with esophageal cancer. JAMA Netw Open. (2020) 3:e2013952. doi: 10.1001/jamanetworkopen.2020.13952 32870311 PMC7489848

[5] Sahgal A Chang JH Ma L Marks LB Milano MT Medin P. Spinal cord dose tolerance to stereotactic body radiation therapy. Int J Radiat Oncol Biol Phys. (2021) 110:124–36. doi: 10.1016/j.ijrobp.2019.09.038 31606528

[6] Costa S Reagan MR. Therapeutic irradiation: consequences for bone and bone marrow adipose tissue. Front Endocrinol (Lausanne). (2019) 10:587. doi: 10.3389/fendo.2019.00587 31555210 PMC6727661

[7] Sturgeon KM Mathis KM Rogers CJ Schmitz KH Waning DL. Cancer- and chemotherapy-induced musculoskeletal degradation. JBMR Plus. (2019) 3:e10187. doi: 10.1002/jbm4.10187 30918923 PMC6419610

[8] Voroney JP Hope A Dahele MR Purdie TG Franks KN Pearson S. Chest wall pain and rib fracture after stereotactic radiotherapy for peripheral non-small cell lung cancer. J Thorac Oncol. (2009) 4:1035–7. doi: 10.1097/JTO.0b013e3181ae2962 19633478

[9] Li Y Behr S. Acute findings on FDG PET/CT: key imaging features and how to differentiate them from Malignancy. Curr Radiol Rep. (2020) 8:22. doi: 10.1007/s40134-020-00367-x 32953250 PMC7486592

[10] Zhong X Li J Zhang L Lu B Yin J Chen Z. Characterization of insufficiency fracture and bone metastasis after radiotherapy in patients with cervical cancer detected by bone scan: role of magnetic resonance imaging. Front Oncol. (2019) 9:183. doi: 10.3389/fonc.2019.00183 30984616 PMC6447664

[11] Barney CL Scoville N Allan E Ayan A DiCostanzo D Haglund KE. Radiation dose to the thoracic vertebral bodies is associated with acute hematologic toxicities in patients receiving concurrent chemoradiation for lung cancer: results of a single-center retrospective analysis. Int J Radiat Oncol Biol Phys. (2018) 100:748–55. doi: 10.1016/j.ijrobp.2017.11.025 PMC7193687 29413286

[12] Kang YM Chao TF Wang TH Hu YW. Increased risk of pelvic fracture after radiotherapy in rectal cancer survivors: A propensity matched study. Cancer Med. (2019) 8:3639–47. doi: 10.1002/cam4.2019.8.issue-8 PMC6639197 31104362

[13] Choi J Oh JY Lee YS Min KH Hur GY Lee SY. P3.15-19 risk factors for osteoporosis in lung cancer patients. J Thorac Oncol. (2018) 13:S998. doi: 10.1016/j.jtho.2018.08.1895

[14] Ebstein E Brocard P Soussi G Khoury R Forien M Khalil A. Burden of comorbidities: Osteoporotic vertebral fracture during non-small cell lung cancer - the BONE study. Eur J Cancer. (2024) 200:113604. doi: 10.1016/j.ejca.2024.113604 38340385

[15] Al-Bashaireh AM Haddad LG Weaver M Chengguo X Kelly DL Yoon S. The effect of tobacco smoking on bone mass: an overview of pathophysiologic mechanisms. J Osteoporos. (2018) 2018:1206235. doi: 10.1155/2018/1206235 30631414 PMC6304634

[16] Ikuta S Shoshihara N Minami S Yasuoka H Takahara K Okamoto Y. A case of radiation-associated vertebral compression fracture mimicking solitary bone metastasis of lung cancer. J Med cases. (2023) 14:293–8. doi: 10.14740/jmc4133 PMC10482597 37692366

[17] Crombag LM Reij E Phernambucq E Senan S Postmus P. Abstracts. O13.05 vertebral collapse after combined modality for locally advanced lung cancer: metastases, osteoporosis, or complication of therapy? A report of 4 cases. J Thorac Oncol. (2011) 6:S39–S902.

[18] Aguilera TA Trakul N Shultz D Maxim PG Diehn M Loo BW. Vertebral fractures after stereotactic ablative radiation therapy of lung tumors. Int J Radiat Oncology Biology Phys. (2014) 90:S160–1. doi: 10.1016/j.ijrobp.2014.05.652 25015205

[19] Brouns A van Veelen A Veerman GDM Steendam C Dursun S van der Leest C. Incidence of bone metastases and skeletal-related events in patients with EGFR-mutated NSCLC treated with osimertinib. JTO Clin Res Rep. (2023) 4:100513. doi: 10.1016/j.jtocrr.2023.100513 37168878 PMC10165134

[20] Shapiro CL Van Poznak C Lacchetti C Kirshner J Eastell R Gagel R. Management of osteoporosis in survivors of adult cancers with nonmetastatic disease: ASCO clinical practice guideline. J Clin Oncol. (2019) 37:2916–46. doi: 10.1200/JCO.19.01696 31532726

[21] Gierloff M Reutemann M Gulses A Niehoff P Wiltfang J Acil Y. Effects of zoledronate on the radiation-induced collagen breakdown: a prospective randomized clinical trial. Clin Transl Oncol. (2015) 17:454–61. doi: 10.1007/s12094-014-1257-8 25425023

[22] Hendriks LE Hermans BC van den Beuken-van Everdingen MH Hochstenbag MM Dingemans AM. Effect of bisphosphonates, denosumab, and radioisotopes on bone pain and quality of life in patients with non-small cell lung cancer and bone metastases: A systematic review. J Thorac Oncol. (2016) 11:155–73. doi: 10.1016/j.jtho.2015.10.001 26718881

[23] Bozzo A Deng J Abbas U Bhasin R Deodat M Wariach S. Which bone-modifying agent is associated with better outcomes in patients with skeletal metastases from lung cancer? A systematic review and network meta-analysis. Clin Orthop Relat Res. (2021) 479:2047–57. doi: 10.1097/CORR.0000000000001749 PMC8373570 33835092

[24] Gumusay O Huppert LA Behr SC Rugo HS. The role of percutaneous vertebral augmentation in patients with metastatic breast cancer: Literature review including report of two cases. Breast. (2022) 63:149–56. doi: 10.1016/j.breast.2022.03.016 PMC8991318 35397256

