# Supplementary material for: Case report: Chest radiotherapy-induced vertebral fractures in lung cancer patients: a case series and literature review
Source: Front Oncol. 2025 Feb 3;15:1438120. doi: 10.3389/fonc.2025.1438120 (PMC11830587; doi:10.3389/fonc.2025.1438120)
Supplement: Supplementary file 1 [file DataSheet1.pdf]

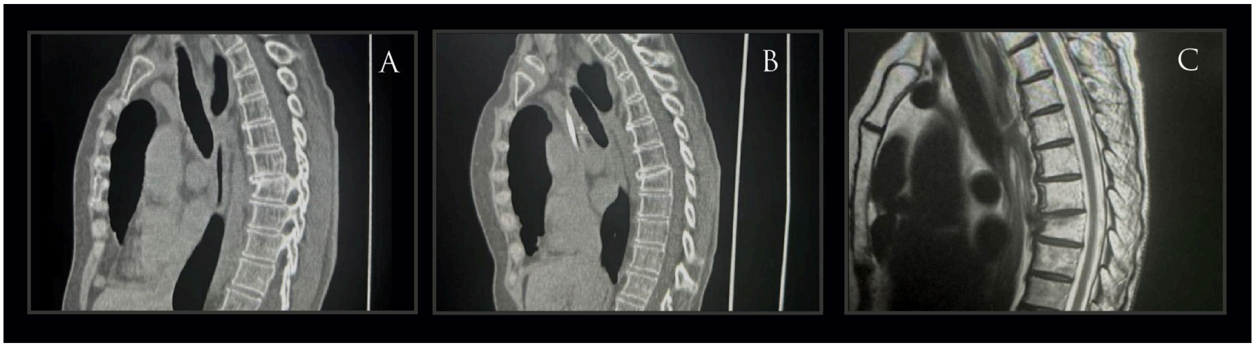

Supplementary Figure 1: Imaging of Case 3. A) CT scan conducted prior to radiotherapy, revealing no evidence of vertebral fractures. B) Follow-up CT post-radiotherapy, depicting a compression fracture in the T8 vertebra. C) MRI showing the presence of the T8 vertebral fracture.

	**Published Reports**		
	**Ikuta et al. [16]**	**Crombag et al. [17]**	**Aguilera et al. [18]**
**Number of patients**	1	4	7
**Age, years**	57	59-68	64-89
**Sex**	Male	Female (4)	Female (6), Male (1)
**Risk factors**	Heavy smoker<br><br>Osteopenia	Osteopenia (3)	Osteopenia (7)
**Histologic type**	Squamous cell carcinoma	NSCLC (3), SCLC (1)	-
**Stage**	IIIA	Locally Advanced	-
**Tumor location**	Right middle lobe	-	-
**Initial treatment**	CBP/paclitaxel + durvalumab	-	-
**RT Regimen**	60 Gy/30 fractions	50 Gy - 66 Gy	20 Gy - 54 Gy (SBRT)
**Dose per fraction**	2 Gy	-	12.5 Gy - 30 Gy
	Concurrent	Concurrent (3), Sequential (1)	-
**Vertebrae w/fracture**	T7	-	Thoracic
**Time from RT to fracture**	12 month	0.5-48 months	-
**Mean Vertebral Radiation Dose (cGy)**	-	29.7 - 53.3 Gy	-
**Maximum Vertebral Radiation Dose (cGy)**	-	47.3 - 68.3 Gy	-
**Type of fracture**	Compression fracture	Compression Fracture	Compression Fracture
**Fracture treatment**	Surgically controlled	Vertebroplasty (1)	-

Supplementary Table 1: Patient characteristics of published cases (One Case Report and Two Abstracts) of patients with radiotherapy-induced vertebral fractures. RT: Radiotherapy, NSCLC: Non-Small Cell Lung Cancer, SCLC: Small Cell Lung Cancer, -: not reported.
